# Early menarche and current nutritional status of the adolescents from a birth cohort

**DOI:** 10.61622/rbgo/2025rbgo28

**Published:** 2025-07-15

**Authors:** Isabela Carolyne de Melo Costa, Maria Teresa Seabra Soares de Britto e Alves, Ana Cleide Vieira, Camila Maria Santos de Sá, Natália Carvalho Fonsêca, Jéssica Mendes Costa de Freitas Santos, Judith Rafaelle Oliveira Pinho, Deysianne Costa das Chagas

**Affiliations:** 1 Universidade Federal do Maranhão São Luís MA Brazil Universidade Federal do Maranhão, São Luís, MA, Brazil.; 2 Escola de Saúde Pública do Maranhão São Luís MA Brazil Escola de Saúde Pública do Maranhão, São Luís, MA, Brazil.

**Keywords:** Menarche, Nutritional status, Adolescent, Birth cohort, Birth weight, Weight gain, Body Mass Index

## Abstract

**Objective:**

To assess the association between early menarche and the nutritional status of the adolescents from the RPS - Brazilian Birth Cohorts Consortium (Ribeirão Preto, Pelotas and São Luís) birth cohort in São Luís, Maranhão.

**Methods:**

A longitudinal study with data from the first and third follow-up of the cohort was conducted. A total of 1,225 adolescents aged 18 to 19 years were surveyed and analyzed for age at menarche, defined as early if <12 years old, and for the variable body mass index (BMI) for age, classified into z-scores by sex, as underweight (z-score <-2), adequate weight (z-score ≥-2 and ≤+1), and excess weight (z-score >+1). Directed acyclic graphs were constructed using the DAGitty^®^ program to select confounding variables for adjustment. Multinomial logistic regression adjusted for confounding variables such as parental obesity (yes or no), skin color (white or non-white), and birth weight (low birth weight, adequate birth weight, and high birth weight) was used to assess the association between early menarche and nutritional status. Statistical analyses were performed using STATA 15.0 software.

**Results:**

Out of the 1,225 adolescents investigated, 32.6% experienced early menarche, and the majority had a BMI appropriate for their age (75.2%). Among adolescents with early menarche, 28.3% were classified as excess weight for their age. Multinomial logistic regression revealed an association between early menarche and excess weight (OR = 1.80; 95% CI = 1.21-2.69; p-value = 0.004).

**Conclusion:**

Thus, the results indicate an association between the occurrence of early menarche and excess weight in the investigated adolescents.

## Introduction

The onset of menstruation is a milestone that serves as a biological and social measure of the healthy transition from childhood to early adulthood.^(1)^ Menarche, the first menstrual period, is therefore just a single event in the combination of physical changes that make up puberty, occurring at the end of this process.^(2)^

Puberty consists of stages involving breast development, pubic hair growth and menarche. The characteristic changes of puberty are triggered and regulated by complex physiological mechanisms influenced by various factors, such as hormonal, socioeconomic, psychosocial, nutritional, genetic and, environmental factors, which interfere with the growth and development process.^(3,4)^

Menarche occurs at different ages in different contexts. Nevertheless, scientific literature points to the increasingly earlier occurrence of menarche in recent years around the world, a phenomenon known as the secular trend in the age of menarche.^(5)^ In Brazil, there have been few studies looking at the temporal trend in the age of menarche; however, it seems that the country follows the global trend.^(6-8)^ Castilho et al.^(6)^ evaluated the age at menarche in adolescents in the city of Campinas, São Paulo, in two distinct periods, 2001 and 2010, and observed an advancement of menarche by 3.2 months between these periods. Costa et al.,^(8)^ in their cross-sectional study conducted in the city of São Luís, Maranhão, when comparing the age at menarche between girls and their mothers, observed a decreasing trend in the age at menarche of mother-daughter pairs.

According to the scientific literature, improvements in living conditions and nutrition, educational, environmental, socioeconomic aspects are possible factors that influence the early onset of menarche.^(3,4,6,8)^ It is believed that early menarche can lead, in adulthood, to some health outcomes such as changes in nutritional status and height, insulin resistance, chronic non-communicable diseases, breast cancer, psychological problems, early onset of sexual relations, teenage pregnancy, sexually transmitted diseases, use of illicit drugs, smoking and alcohol consumption.^(1,3,4,9-12)^

There are reports in the scientific literature of a higher prevalence of excess weight in girls who experienced early menarche. However, there is no consensus as to whether early menarche favors excess weight or whether the excess weight present in childhood predicts early sexual maturation. Therefore, the relationships between early menarche and excess weight have been widely investigated in order to clarify the cause and/or consequence effect between them.^(12-18)^

Furthermore, some studies indicate the relationship between birth weight and early menarche. According to the literature, adolescents born with low birth weight have a higher risk of earlier menarche, which can be explained by multifactorial mechanisms such as the regulation of intrauterine and infant growth.^(19,20)^

In relation to birth weight and nutritional status in adolescence, the literature points to controversial results by showing that extremes of birth weight (low birth weight and high birth weight) are sometimes unrelated and sometimes indicated as predictors of future obesity risk.^(21,22)^

Given the various implications associated with the early onset of menarche, this study aimed to investigate the association between early menarche (<12 years) and nutritional status, measured by the BMI-for-age indicator, in adolescents participating in a birth cohort in São Luís, Maranhão, Brazil. For this purpose, a *Directed Acyclic Graph* (DAG) was constructed to select the minimal variables for adjusting the multivariate regression model.

## Methods

This is a longitudinal study conducted based on data from birth and the third follow-up of the mixed birth cohort with adolescents born in the city of São Luís, Maranhão, Brazil. The study, titled ‘Lifelong Determinants of Obesity, Precursors of Chronic Diseases, Human Capital, and Mental Health: a contribution from Brazilian birth cohorts to the Brazilian Unified National Health System (SUS),’ was developed by the RPS Cohort Consortium composed of the *Universidade Federal do Maranhão* (UFMA), the *Faculdade de Medicina de Ribeirão Preto* (Universidade de São Paulo), and the *Universidade Federal de Pelotas* (UFPel).

The RPS cohort in São Luís was conducted in three follow-ups (birth, school age, and adolescence). In the first follow-up, the perinatal study was conducted in ten hospitals, both public and private, in the city of São Luís, from March 1997 to February 1998, using a sampling base of one in every seven births, resulting in 2,541 births being included. Multiple births, stillbirths, and deaths in the first year of life were excluded, resulting in a sample of 2,378 births. Subsequently, the second follow-up took place between 2005 and 2006, during which 805 children from the initial sample, aged 7 to 9 years old, were evaluated. In the third follow-up, conducted in 2016, adolescents aged 18 and 19 were studied. All participants in this follow-up were sought out in the four Military Enlistment Boards in São Luís, in the 2014 School Census, and at universities. The adolescents identified as participants of the cohort were invited to attend the follow-up, totaling 687 participants. However, due to the small sample size of this follow-up, there was a need to expand it. To increase its size and prevent sample losses, new participants born in São Luís in the year 1997 and who were not initially part of the cohort were randomly selected. These were identified through school or university registration and military enlistment, between January and November 2016, totaling 1,828 new adolescents and a final sample of 2,515 adolescents. After selecting the participants, interviews were conducted with questionnaires, and body composition was assessed. The new participants were questioned about birth-related variables to supplement the information.^(23)^ This study included all female adolescents (n=1,327) from the third follow-up sample of the birth cohort under study. From the initial sample population, 33 adolescents were excluded due to missing information on menarche age, 65 due to missing information on pregnancy status, and 4 due to the use of plaster bandage at the time of anthropometric measurements acquisition, which could interfere with obtaining anthropometric measurements.

**Figure 1:**
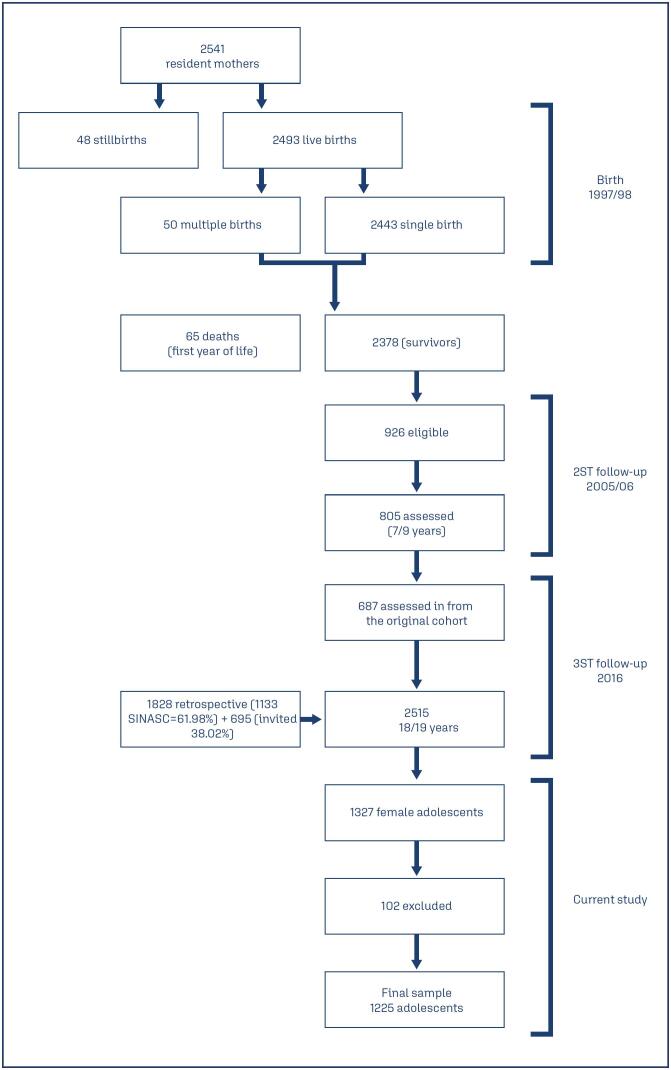
Flowchart of the RPS birth cohort

For the sample size calculation, a prevalence of 30% was assumed for early menarche and a prevalence difference of 10% between the exposed and unexposed groups. The power of the test was set at 80, and the probability of a type I error was set at 0.05. This resulted in a total of 588 adolescents. To compensate for the data loss, the final required sample size was 647 adolescents. The final sample consisted of 1,225 adolescents (Figure 1).

The research data were collected in the city of São Luís, Maranhão, by a team of properly trained researchers. Participants answered standardized and structured forms specifically designed for this study, addressing questions about individual, socioeconomic, family, demographic, health, and lifestyle aspects. Anthropometric and body composition variables were measured by a trained team. The question related to alcohol consumption experience was collected through a self-administered questionnaire. The data was collected and digitized using the Research Electronic Data Capture (Redcap^®^) program.

The exposure variable analyzed was the age at menarche. For this, adolescents answered the question "How old were you when you had your first menstrual period?" and early menarche was considered to have occurred at age <12 years. This cut-off point was defined considering its use by several studies and based on a pioneering and nationally representative study conducted by Barros et al.^(24)^ that estimated the median age of menarche at 12.4 years.^(10-14)^

The outcome variable analyzed was nutritional status, measured by BMI-for-age. The weight of the adolescents, in kilograms, was measured using the air displacement plethysmography technique, utilizing the BodPod^®^ Gold Standard device from the COSMED brand. Nutritional status was classified according to the guidelines for the collection and analysis of anthropometric data in health services from the Brazilian Ministry of Health, based on the body mass index (BMI) for age and sex in z-scores, and grouped into three categories: underweight, adequate weight, and excess weight (overweight and obese), with cut-off points at <-2, between ≥-2 and ≤+1, and >+1, respectively.^(25)^

Among the covariates evaluated in this study were: age in years (18 and 19 years), skin color (white and non-white), marital status (without partner and with partner), schooling in years (0 to 8 years, 9 to 11 years, and 12 or more years), head of the family (parents and others), schooling of the family head in years (0 to 8 years, 9 to 11 years, 12 or more years), parents separated/divorced (yes and no), economic class (A/B, C, and D/E) according to the Brazil Economic Classification,^(26)^ smoking (yes and no), alcohol consumption in the last 12 months (yes and no), level of physical activity (sedentary, low, and moderate/high) according to the Self Administration Physical Activity Checklist - SAPAC^®^,^(27)^ satisfaction with health (very dissatisfied/dissatisfied, regular, and satisfied/very satisfied), parental obesity (yes and no), birth weight (low birth weight, adequate birth weight, and high birth weight), current work (yes and no), eating habits (according to the information from the Food Frequency Questionnaire), and number of children (1 and 2).

Data analysis was conducted in three stages. Initially, a descriptive analysis of the data was carried out to estimate the frequency of early menarche and the nutritional status of the adolescent sample. Absolute and relative frequencies of sociodemographic, economic, lifestyle habits, and satisfaction with health were calculated according to nutritional status.

**Figure 2:**
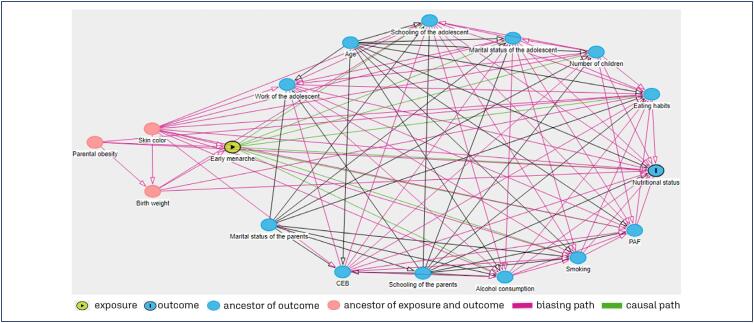
Directed Acyclic Graph to assess the association between early menarche and nutritional status of the adolescents from a birth cohort

CEB: Brazil economic classification; PAF: level of physical activity

Subsequently, in order to select confounding variables for adjustment, an extensive literature search was carried out to identify the variables, and Directed Acyclic Graphs (DAG) were constructed using the DAGitty^®^ program, version 3.0, available free of charge from http://www.dagitty.net. DAGs provide simple and useful graphs for delineating and understanding confounding factors and potential sources of bias in exposure-outcome relationships by representing causal relationships between variables, with arrows pointing from cause to effect.^(28)^ A graph was therefore constructed to assess the association between early menarche and the nutritional status of the adolescents (Figure 2). After constructing the graph, the variables selected for model adjustment were: parental obesity, skin color and birth weight.

Finally, multivariate regression analysis was conducted to examine the relationship between the selected variables for model adjustment. Multinomial logistic regression was used to estimated odds ratios and 95% confidence intervals (95%CI). The statistical tests were performed using Stata software, version 15.0.

The present study was approved by the Research Ethics Committee of the Universidade Federal do Maranhão (approval no. 1.302.489 dated October 29, 2015, and *Certificado de Apresentação de Apreciação Ética* no. 49096315.2.0000.5086), and adolescents who agreed to participate in the study signed the Informed Consent Form (ICF).

## Results

The sample, composed of 1,225 adolescents, had a median age of menarche of 12 years. Among them, 399 (32.6) experienced early menarche, and 263 (21.5) were classified as excess weight. Among the girls with early menarche, 113 (28.3) were classified as having excess weight (Table 1).

**Table 1:** Early menarche and nutritional status of the adolescents from a birth cohort

Early menarche	Nutritional status		
	Underweight	Adequate weight	Excess weight
	n(%)	n(%)	n(%)
Yes	9(2.3)	277(69.4)	113(28.3)
No	31(3.7)	645(78.1)	150(18.2)

The majority of the adolescents had adequate nutritional status (75.2%), and among these, most were 18 years old (68.0%), were non-white (80.1%), lived without a partner (96.8%), had 9 to 11 years of schooling (89.5%), had one of their parents as the head of the family (75.8%), with the family head having 9 to 11 years of schooling (52.6%), were not separated/divorced (51.3%), belonged to class C (47.2%), were non-smokers (59.3%), had consumed alcohol in the last 12 months (50.2%), were sedentary (64.4%), and reported being satisfied/very satisfied with their health (47.8%) (Table 2).

**Table 2:** Nutritional status and demographic and socioeconomic characteristics, lifestyle habits and satisfaction with health of the adolescents from a birth cohort

Variables		Nutritional status			Total
		Underweight	Adequate weight	Excess weight	
		n(%)	n(%)	n(%)	n(%)
Demographic and socioeconomic					
Age (years)					
	18 years	25(62.5)	627(68.0)	170(64.6)	822(67.1)
	19 years	15(37.5)	295(32.0)	93(35.4)	403(32.9)
Skin color*					
	White	6(15.8)	183(19.9)	62(23.8)	251(20.6)
	Non-white	32(84.2)	735(80.1)	199(76.2)	966(79.4)
Marital status					
	Without partner	39(97.5)	892(96.8)	242(92.0)	1,173(95.8)
	With partner	1(2.5)	30(3.2)	21(8.0)	52(4.2)
Schooling (years)**					
	0 to 8 years	4(10.0)	31(3.4)	8(3.1)	43(3.5)
	9 to 11 years	32(80.0)	822(89.5)	231(88.5)	1,085(89.0)
	12 or more years	4(10.0)	65(7.1)	22(8.4)	91(7.5)
Head of the family					
	Parents	30(75.0)	699(75.8)	182(69.2)	911(74.4)
	Others	10(25.0)	223(24.2)	81(30.8)	314(25.6)
Schooling of the family head (years)***					
	0 to 8 years	10(28.6)	242(29.0)	67(28.2)	319(28.8)
	9 to 11 years	17(48.6)	440(52.6)	120(50.4)	577(52.0)
	12 or more years	8(22.8)	154(18.4)	51(21.4)	213(19.2)
Parents separated/divorced					
	Yes	20(50.0)	449(48.7)	129(49.1)	598(48.8)
	No	20(50.0)	473(51.3)	134(50.9)	627(51.2)
Economic class****					
	A/B	7(20.6)	229(27.7)	63(27.0)	299(27.4)
	C	16(47.1)	390(47.2)	120(51.5)	526(48.1)
	D/E	11(32.3)	207(25.1)	50(21.5)	268(24.5)
Lifestyle habits and satisfaction with health Smoking*****					
	Yes	0(0.0)	22(40.7)	4(25.0)	26(37.1)
	No	0(0.0)	32(59.3)	12(75.0)	44(62.9)
Alcohol consumption (last 12 months)******					
	Yes	20(50.0)	459(50.2)	154(59.2)	633(52.1)
	No	20(50.0)	456(49.8)	106(40.8)	582(47.9)
Level of physical activity*******					
	Sedentary	29(74.4)	593(64.4)	151(57.8)	773(63.3)
	Low	7(17.9)	110(11.9)	31(11.9)	148(12.1)
	Moderate/High	3(7.7)	218(23.7)	79(30.3)	300(24.6)
Satisfaction with health					
	Very dissatisfied/dissatisfied	11(27.5)	136(14.8)	63(23.9)	210(17.1)
	Regular	15(37.5)	345(37.4)	98(37.3)	458(37.4)
	Satisfied/Very satisfied	14(35.0)	441(47.8)	102(38.8)	557(45.5)

N total: 1217*, 1219**, 1109***, 1093****, 70*****, 1215******, 1221*******

In the multinomial logistic regression between menarche and nutritional status by age, adjusted for parental obesity, skin color and birth weight, an association was observed between early menarche and excess weight (OR = 1.80; 95%CI = 1.21-2.69; p-value = 0.004) (Table 3).

**Table 3:** Adjusted multinomial logistic regression for the association between early menarche and excess weight among adolescents from a birth cohort

Variables		OR	95% CI	p-value
Early menarche				
	No	1	-	-
	Yes	1.80	1.21 – 2.69	0.004
Parental obesity				
	No	1	-	-
	Yes	8.81	2.24 – 34.61	0.002
Skin color				
	White	1	-	-
	Non-white	0.71	0.46 – 1.10	0.134
Birth weight				
	Adequate	1	-	-
	Low	0.55	0.26 – 1.16	0.117
	High	2.17	1.13 – 4.16	0.019

## Discussion

In this study, we investigated the association between early menarche and nutritional status of 1,225 adolescents aged 18 to 19 years. The results indicated an association between early menarche and excess weight in the investigated adolescents, regardless of birth weight. It is worth noting that approximately one-third of them experienced menarche at an early age (<12 years). Additionally, although the majority had an adequate nutritional status, approximately a quarter of these adolescents had excess weight.

The occurrence of early menarche in this study illustrates the global trend of early menarche, since in populations studied in previous years, the age of menarche was later in Brazil and worldwide.^(4,6,7)^

Nutritional status measured through anthropometric measurements is a reflection of the balance between nutrient intake and the body's energy expenditure.^(25)^ However, this relationship is influenced by other factors, as it represents the economic, social, family, ideological, and behavioral structure in which the individual is inserted.^(29)^

Excess weight constitutes a risk factor for chronic non-communicable diseases and promotes long-term impact by reducing physical health levels.^(30)^ According to the literature, studies indicate that obese adolescents tend to remain obese in adulthood, with effects on the healthcare system, leading to increased healthcare expenditure.^(31)^

In this study, adolescents who experienced early menarche had a higher risk of having excess weight when compared to those who experienced menarche at an adequate age. This result is also supported by other studies that indicated early menarche as a factor contributing to the higher prevalence of overweight and obesity in adulthood.^(12-14)^ Gonçalves et al.^(14)^ when investigating reproductive factors associated with overweight in adulthood found a positive association between overweight and early age of menarche, with the prevalence of overweight being 12.4% lower in women with menarche at ≥12 years. Theodoro et al.^(12)^ observed that women with early menarche had a 75% higher prevalence of obesity compared to women with menarche at a later age.

Some studies have not found an association between early menarche and excess weight or have indicated an attenuation of this association when adjusted for weight during childhood. Araújo et al.^(18)^ and Bratberg et al.^(32)^ found that excess weight did not differ between girls with early menarche and their peers, indicating that there is an association between these variables only when there is an increase in adiposity prior to menarche. Freedman et al.^(33)^ and Must et al.^(34)^ observed an association between early menarche and excess weight, but this was attenuated when adjusted for weight and BMI in childhood. Nevertheless, as in the current study, the relationship between early menarche and excess weight has been reported by several studies, suggesting an association independent of nutritional status prior to menarche.^(13,14,16,17)^ Some factors may have contributed to the results found by these authors, namely, sample size, loss to follow-up, and lack of standardization in the measurement of anthropometric measures.

The scientific literature suggests that high birth weight can be a risk factor for future excess weight.^(21,22)^ However, this study demonstrated that early menarche constituted an increased risk factor for excess weight, regardless of birth weight.

This relationship can be explained by the increased production of gonadal hormones, which are responsible for physiological changes in body composition during this phase of life. One of the proposed explanations is that early menarche results in longer exposure to estrogen and adrenal steroids, which are responsible for maintaining adiposity.^(35)^ However, the mechanism by which the predictors of precocity influence menarche, and how this, in turn, affects the development of excess weight, remains unclear.

According to the literature, excess weight appears to be not only a consequence of early menarche but also a precursor to it. In this study, the causative effects of early menarche were not investigated, but this result has been observed in previous studies.^(6,15,24)^ Furthermore, scientific literature has suggested that BMI in childhood may be a determinant of the age of menarche and that changes in nutritional status in childhood may persist into adulthood, affecting and being affected by sexual maturation and, consequently, the age of menarche.^(15-17,33)^ This emphasizes the importance of monitoring nutritional status in childhood and adolescence, because, as well as influencing sexual maturation at this time of life, nutritional deviations can continue into adulthood.

The strengths of the study include the sample size, standardized data collection, and the longitudinal design, providing information at different time points and enabling the possibility of an analysis considering confounding factors at two distinct stages of the life cycle of the surveyed adolescents. Our limitations include the lack of data on nutritional status at the time of menarche and during childhood, which limits the understanding of the trajectory of nutritional status over time and prevents us from analyzing the development and/or maintenance of excess weight. On the other hand, the analysis model used allows us to infer an independent association between early menarche and excess weight. We also acknowledge the possibility of memory bias in recalling the age of menarche and birth weight. However, given the significant relevance of this milestone in a woman's life, this method is considered appropriate for providing accurate information on the age of menarche, as well as birth weight, which is a salient piece of information that mothers can easily recall.

## Conclusion

In this study, an association was found between the occurrence of early menarche and excess weight in adolescents from a birth cohort in São Luís, Maranhão. The menarche occurred early in approximately one-third of the adolescents. Most of the adolescents were classified as having an adequate nutritional status, although one in five adolescents in the cohort was classified as excess weight. Finally, the results of this study point to the importance of public policies and health education programs addressing the impacts of early menarche on the lives of adolescents, including aspects related to healthy lifestyle habits and regular health monitoring. Although it is not possible to change the time of occurrence of menarche, early intervention in potential outcomes can help prevent excess weight and other chronic non-communicable diseases, thus improving the quality of life of this population.
